# The Toxin Gene *tdh2* Protects *Vibrio parahaemolyticus* from Gastrointestinal Stress

**DOI:** 10.3390/microorganisms13081788

**Published:** 2025-07-31

**Authors:** Qin Guo, Jia-Er Liu, Lin-Xue Liu, Jian Gao, Bin Xu

**Affiliations:** School of Food and Biological Engineering, Jiangsu University, Zhenjiang 212013, China; 2222218110@stmail.ujs.edu.cn (J.-E.L.); 2212318052@stmail.ujs.edu.cn (L.-X.L.); 2222418077@stmail.ujs.edu.cn (J.G.)

**Keywords:** foodborne pathogen, environmental stress, gene knockout, regulation mechanism

## Abstract

*Vibrio parahaemolyticus* is a major foodborne pathogen worldwide, responsible for seafood-associated poisoning. Among its toxin genes, *tdh2* is the most critical. To investigate the role of *tdh2* in *V. parahaemolyticus* under gastrointestinal conditions, we constructed *tdh2* deletion and complementation strains and compared their survival under acid (pH 3 and 4) and bile stress (2%). The results showed that *tdh2* expression was significantly upregulated under cold (4 °C) and bile stress (0.9%). Survival assays and PI staining revealed that the *tdh2* mutant strain (VP: △*tdh2*) was more sensitive to acid and bile stress than the wild-type (WT), and this sensitivity was rescued by *tdh2* complementation. These findings suggest that *tdh2* plays a protective role in enhancing *V. parahaemolyticus* tolerance to acid and bile stress. In the VP: △*tdh2* strain, seven genes were significantly upregulated and six were downregulated as a result of *tdh2* deletion. These genes included *VPA1332* (*vtrA*), *VPA1348* (*vtrB*), *VP2467* (*ompU*), *VP0301* and *VP1995* (ABC transporters), *VP0527* (*nhaR*), and *VP2553* (*rpoS*), among others. Additionally, LC-MS/MS analysis identified 12 differential metabolites between the WT and VP: △*tdh2* strains, including phosphatidylserine (PS) (17:2 (9Z,12Z) /0:0 and 20:1 (11Z) /0:0), phosphatidylglycerol (PG) (17:0/0:0), flavin mononucleotide (FMN), and various nucleotides. The protective mechanism of *tdh2* may involve preserving cell membrane permeability through regulation of *ompU* and ABC transporters and enhancing electron transfer efficiency via regulation of *nhaR*. The resulting reduction in ATP, DNA, and RNA synthesis—along with changes in membrane permeability and electron transfer due to decreased FMN—likely contributed to the reduced survival of the VP: △*tdh2* strain. Meanwhile, the cells actively synthesized phospholipids to repair membrane damage, leading to increased levels of PS and PG. This study provides important insights into strategies for preventing and controlling food poisoning caused by *tdh^+^ V. parahaemolyticus*.

## 1. Introduction

*Vibrio parahaemolyticus* is a Gram-negative, halophilic, pathogenic bacterium widely distributed in seafood, salted foods, seawater, and seafloor sediments. Excessive intake (more than 10^6^ CFU/g) of *V. parahaemolyticus* by humans will cause acute gastroenteritis (e.g., diarrhea) [1]. *V. parahaemolyticus* is the one of the causes associated with outbreaks of seafood-associated poisoning reported in many countries, including the United States, France, Italy, Australia, Brazil, New Zealand, India, South Korea, Malaysia, Chile, and especially in China in recent years [2,3]. From 2013 to 2022, 23,818 cases of *V. parahaemolyticus* diarrhea were documented in China and 10.83% of acute diarrhea in China was caused by *V*. *parahaemolyticus* between 2009 and 2018, 78.20% of which occurred between July and September [4,5,6]. A total of 22.8% of foodborne diseases in Chile from 2016 to 2022 were caused by *V*. *parahaemolyticus* [7]. A total of 268 persons infected with *V. parahaemolyticus* from oysters in Australia were reported between 2021 and 2022 [8]. These outbreaks belong to serotypes O3:K6, O4:K68, O1: KUT, and O1:K25, which rapidly spread throughout the world in the last 20 years [9].

*V. parahaemolyticus* includes non-pathogenic strains lacking toxin genes and pathogenic strains, which are further classified into pandemic and non-pandemic clones. For non-pathogenic strains, ingestion of more than 10^6^ CFU/g is typically required to cause acute gastroenteritis in humans. However, pathogenic *V. parahaemolyticus* can result in more severe outcomes, particularly in immunocompromised individuals, due to virulence factors such as thermostable direct hemolysin (TDH), TDH-related hemolysin (TRH), urease, and type III secretion system 2 (T3SS2) [10]. Among these, TDH—encoded by *tdh* genes—is regarded as a major virulence factor. Five *tdh* genes have been identified: four chromosomally encoded (*tdh1*, *tdh2*, *tdh3*, and *tdh4*) and one plasmid-borne (*tdh5*) [11]. These genes share over 96.7% sequence identity and exhibit similar biological functions. Among them, *tdh2* (VPA1314) is the predominant determinant of TDH activity due to its significantly higher expression compared to the other *tdh* genes [12]. *tdh2* is located on an 80-kb pathogenicity island (Vp-PAI) on chromosome II in pathogenic *V. parahaemolyticus* strains [13].

TDH is a water-soluble monomer composed of 165 amino acid residues forming a compact β-sandwich core structure. Its C-terminal region (CTR), tethered to the β-sandwich domain via an intramolecular disulfide bond, facilitates TDH oligomerization prior to membrane binding [14]. As a pore-forming toxin, TDH forms tetramers that generate pores approximately 2 nm in diameter in erythrocyte and epithelial cell membranes, allowing the flow of ions and fluids—likely contributing to diarrhea [15]. TDH channel formation in phospholipid bilayers depends on the presence of phospholipids rather than cholesterol or receptor proteins [16]. TDH causes hemolysis on Wagatsuma blood agar, known as the Kanagawa phenomenon (KP), and is involved in the hemolytic, cardiotoxic, cytotoxic, and enterotoxic effects of *V. parahaemolyticus*. Its cytotoxicity is lipid raft-dependent in epithelial cells [17].

The expression of *tdh* and production of TDH increase under environmental stress. Bile is considered one of the key factors that significantly induces *tdh* expression and TDH production, with the overproduced toxins enabling the bacterium to acquire nutrients from host cells [18]. High or low temperatures and high salinity also enhance *tdh* expression [19]. Other environmental signals—such as high concentrations of sugars, inorganic or organic acids, the monoamine ethanolamine, and ethanol in the growth medium—can also affect TDH production in *V. parahaemolyticus* [20,21]. Elevated *tdh* expression under stress conditions is associated with regulatory elements such as *calR*, *hfq*, *H-NS*, the *toxRS* operon, and *toxRS*-like transcriptional regulators *vtrA* and *vtrB*, particularly *toxRS* [12,22,23,24,25].

Pathogenic *V. parahaemolyticus* strains harboring virulence genes may exhibit different stress tolerance levels compared to non-pathogenic strains [26]. Strains carrying *tdh* genes demonstrate greater competitive colonization ability in the mouse intestinal tract. However, *tdh* was not among the 230 genes identified as significantly contributing to intestinal colonization [27]. To date, limited information is available regarding the role of *tdh*, particularly *tdh2*, in environmental adaptability—especially within the intestine.

In this study, we focused on investigating the potential functions and mechanisms of *tdh2* in protecting *V. parahaemolyticus* under environmental stress. Our findings indicate that *tdh2* is essential for the selection and survival of pathogenic *V. parahaemolyticus* within the intestinal environment. This study may offer valuable insights into the prevention and control of foodborne illness caused by *tdh^+^ V. parahaemolyticus*.

## 2. Materials and Methods

### 2.1. Strains, Plasmids, and Growth Conditions

The strains and plasmids used in this study are listed in Table 1. *V*. *parahaemolyticus* strains were routinely cultivated on TCBS agar (Qingdao Hopebiol Biotechnology Company, Qingdao, China) or in Luria-Bertani (LB) broth supplemented with 3% NaCl (LBNB) at 37 °C, with 5 μg/mL chloramphenicol added when necessary. *Escherichia coli* DH5ɑ and S17-1-pir were cultured in LB broth at 37 °C, supplemented with 100 μg/mL ampicillin or 10 μg/mL chloramphenicol, as needed. For solid media, 2% (*w/v*) agar was added.

*V. parahaemolyticus* RIMD2210633 was used to construct the *tdh2* mutant. *E. coli* DH5ɑ was used for plasmid maintenance and manipulation, and *E. coli* S17-1-pir was employed for conjugal plasmid transfer into *V. parahaemolyticus*. Plasmids pDS132 and pACYC184 were used as backbone vectors for gene knockout and for the construction of the *tdh2* complementary strain, respectively.

### 2.2. The Growth Measurement

*V. parahaemolyticus* cultures were grown overnight with shaking at 225 rpm in 5 mL of LBNB at 37 °C. Then, 100 μL of the overnight cultures were transferred into separate tubes containing 5 mL of fresh LBNB and incubated at 37 °C for various durations. At designated time points, the tubes were removed, and both the optical density at 600 nm (OD_600_) and total colony counts were measured.

### 2.3. Response of V. parahaemolyticus to Cold and Bile Stress

*V. parahaemolyticus* strains were cultivated in LBNB at 37 °C with shaking at 225 rpm for 12 h as the control condition. For bile stress treatment, cultures were grown in LBNB at 37 °C for 8 h, followed by the addition of bile salts to a final concentration of 0.9%, and incubated for an additional 4 h. For cold shock treatment, cultures were grown in LBNB at 37 °C for 8 h and then transferred to 4 °C for 4 h. Following bile or cold stress, 20 mL of each culture was centrifuged at 8000× *g* for 2 min at 4 °C (for cold-stress samples) or at room temperature (for control and bile-stress samples). The cell pellets were collected and resuspended in 1 mL of Sample Protector (Beijing Adlai Biotechnology Co., Ltd., Beijing, China) to preserve temporal RNA expression changes. These samples were stored at 4 °C for up to 7 days. When needed, samples were centrifuged at 5000× *g* for 5 min at room temperature, washed twice with RNase-free ddH_2_O to remove the sample protector, and resuspended in 100–200 μL of Tris-EDTA buffer (pH 8.0), adjusting the final bacterial concentration to approximately 10^6^ CFU/mL for RNA extraction (see Section 2.8). *V. parahaemolyticus* cells not subjected to bile or cold stress served as the control. The expression of *tdh2* was then measured by RT-qPCR, as described in Section 2.8.

### 2.4. The Simulation of Gastrointestinal Stress In Vitro

#### 2.4.1. Acid Tolerance

The pH of fasting gastric acid in the human body ranges from approximately 1.5 to 3.5 and increases to 4–5 after eating. To simulate gastric acid conditions (pH 3–4) and passage time (0.5–2 h), we tested the survival of *V. parahaemolyticus* under acid stress in vitro. LBNB was adjusted to pH 3 and 4 using 0.1 mol/L HCl, and the pH was calibrated with a pH meter (PHS-2F, INESA Analytical Instrument Co., Ltd., Shanghai, China) to ensure consistency across experimental groups. *V. parahaemolyticus* strains (WT, VP: △*tdh2*, and C-△*tdh2*) were cultured in 5 mL of LBNB at 37 °C for 12 h with shaking at 225 rpm. The cultures were harvested by centrifugation at 10,000× *g* at 4 °C for 5 min, and the pellets were resuspended in LBNB to an OD_600_ of 0.7 (approximately 10^8^ CFU/mL). Then, 100 μL of each suspension was transferred into separate tubes containing 5 mL of LBNB adjusted to pH 3 or 4. The tubes were incubated at 37 °C with shaking at 225 rpm, and viable cells were determined at 0.5 and 2 h using the standard plate count method on LB agar containing 3% NaCl. Each experimental group was performed in triplicate. All procedures followed standardized protocols to ensure the stability and reproducibility of the results.

#### 2.4.2. Bile Tolerance

To evaluate the survival of *V. parahaemolyticus* under bile stress in vitro, we used a 2% bile salt concentration and a passage time of 2–4 h. A total of 0.10 g of bile salt (from pig) was weighed, dissolved slowly in 5 mL of LBNB by vortexing, and then filtered through a 0.22 μm membrane filter for use. *V. parahaemolyticus* strains (WT, VP: △*tdh2*, and C-△*tdh2*) were cultured in LBNB at 37 °C for 12 h with shaking at 225 rpm. The cultures were pelleted by centrifugation at 10,000× *g* at 4 °C for 5 min and then resuspended in LBNB containing 2% bile salt to an OD_600_ of 0.7 (approximately 10^8^ CFU/mL). The suspensions were incubated at 37 °C, and samples were collected at 2 and 4 h. The survival rate was calculated as: (colonies on LB agar plate/number of inoculated cells) × 100%**.** Each experimental group was conducted in triplicate. All procedures followed standardized protocols to ensure the consistency and repeatability of the experiment.

### 2.5. SDS-PAGE Analysis of Whole-Cell and Secreted Proteins

Both bacterial and secreted proteins were analyzed using SDS-PAGE. For bacterial protein analysis, 50 μL of overnight cultures of *V. parahaemolyticus* WT and VP: △*tdh2* were inoculated into fresh LBNB and cultured at 37 °C with shaking at 225 rpm for 18 h. Then, 1 mL of the bacterial suspension was transferred into a 1.5 mL centrifuge tube and centrifuged at 10,000 rpm for 5 min. The pellet was resuspended in 50 μL of 2 × SDS loading buffer, boiled at 100 °C for 10 min, and centrifuged again at 10,000 rpm for 10 min. The supernatant was used for electrophoresis using 10% SDS-PAGE.

For secreted protein analysis, 50 mL of overnight cultures of *V. parahaemolyticus* WT and VP: △*tdh2* were centrifuged at 12,000× *g* for 15 min. The supernatants were sterilized by filtration through a 0.22 μm filter and proteins were precipitated with 10% (*v/v*) trichloroacetic acid (TCA) on ice for 60 min. The precipitated proteins were collected by centrifugation at 15,000× *g* for 15 min, washed with cold acetone, dissolved in PBS buffer, and analyzed by 10% SDS-PAGE. Protein concentrations before SDS-PAGE were estimated using a BCA Protein Assay Kit (Beyotime Biotechnology, Beijing, China). Protein bands were visualized using Coomassie Brilliant Blue R-250 staining (Sinopharm Chemical Reagent Co., Ltd., Shanghai, China). SDS-PAGE was performed according to the manufacturer’s instructions.

### 2.6. Construction of V. parahaemolyticus ∆tdh2 Mutant

The ORF of *tdh2* was deleted using splicing overlap extension (SOE) PCR and allelic exchange. Briefly, using the genome of *V. parahaemolyticus* RIMD2210633 as the template, two pairs of primers (1314-A/1314-B and 1314-C/1314-D), homologous to the upstream and downstream regions of the *tdh2* gene, were designed to perform SOE PCR (Table 2), yielding a 758 bp knockout fragment. This fragment was cloned into the suicide vector pDS132, which was designated as pDS132-*tdh2*. The resulting plasmid was transformed into *E. coli* S17-λ-pir and then conjugated into *V. parahaemolyticus* RIMD2210633 via cross-streaking on LB plates. The resulting cultures were washed twice with LB broth and transferred onto TCBS plates containing 5 μg/mL chloramphenicol to selectively isolate *V. parahaemolyticus* harboring pDS132-*tdh2*. Exconjugant colonies were sub-cultured multiple times overnight in LB broth containing 10% sucrose to promote loss of the pDS132-*tdh2* plasmid. Double-crossover deletion mutants, which were sensitive to chloramphenicol, were screened by replica plating on LB-10% sucrose plates and LB plates containing 5 μg/mL chloramphenicol. Suspected deletion mutants were further verified by PCR using primer sets 1314-E/1314-d, *tdh2*-F/*tdh2*-R, and *sacB*-F/*sacB*-R. One confirmed mutant with *tdh2* deletion was designated as VP: ∆*tdh2*.

### 2.7. Construction of the Complemented Strain VP-C-△tdh2

A *V. parahaemolyticus* strain expressing a complemented *tdh2* gene was constructed using a complete *tdh2* expression cassette with pACYC184 as the backbone vector. A 1052 bp DNA fragment containing the promoter, signal peptide, coding sequence (CDS), and terminator of the *tdh2* gene was amplified using primers *tdh*-HB-F/*tdh*-HB-R (Table 2). PCR was performed under the following conditions: 95 °C for 5 min; 29 cycles of 94 °C for 30 s, 55 °C for 30 s, and 72 °C for 1 min; followed by 72 °C for 10 min. The PCR product was cloned into pACYC184 using *SphI* and *BamHI* restriction sites, generating plasmid pACYC184-*tdh2*. The plasmid was then introduced into VP-∆*tdh2* (at a ratio of 1:10) by electroporation (1.5 kV, 15 ms) using a BTX ECM 830 electroporator (Harvard Apparatus, Horiston, MA, USA), yielding the complemented strain VP-C-△*tdh2*. The recombinant strain was verified by PCR (*tdh*-HB-F/*tdh*-HB-R), plasmid extraction, and sequencing. To maintain the stability of the plasmid, chloramphenicol was added to LB agar or LBNB at a final concentration of 5 μg/mL.

### 2.8. RNA Extraction and Real-Time Fluorescence Quantitative PCR (RT-qPCR) Analysis

*V. parahaemolyticus* WT and VP: △*tdh2* strains were cultured overnight, then diluted 1:100 into 5 mL of LBNB and cultured again for 12 h at 37 °C with shaking at 225 rpm. The cultures were centrifuged at 10,000 rpm and the pellets were resuspended in LBNB to a final concentration of 10^6^ CFU/mL for RNA extraction.

Total bacterial RNA was extracted using the EASYspin rapid bacterial RNA extraction kit (Beijing Adlai Biotechnology Co., Ltd., Beijing, China), following the manufacturer’s protocol. RNA quality was assessed by 1% TAE agarose gel electrophoresis, and concentration and purity were determined using a NanoDrop 2000 Nucleic Acid Analyzer (Thermo Fisher Scientific Inc., Waltham, MA, USA) (see Supplementary Data, Figure S1 and Table S1). One microgram of DNase I-treated RNA from the WT and VP: △*tdh2* was reverse transcribed into cDNA using the Sensiscript RT Kit (Takara Biotech, Beijing, China). The resulting cDNA was used as a template for RT-qPCR on the Bio-Rad CFX96 Real-Time PCR Detection System (Bio-Rad, Hercules, CA, USA). A total of 22 genes potentially regulated by *tdh2* were selected based on the literature review. Primer sequences are listed in Table 3. PCR amplification was conducted in a 20 μL reaction containing 10 μL of 2 × SuperReal Premix Plus (Takara Biotech, Beijing, China), 2.0 μL of cDNA, 6.8 μL of sterile water, and 0.6 μL each of forward and reverse primers (Table 3). RNase-free ddH_2_O was used as the negative control. Reaction mixtures were prepared in 8-tube strips on ice. 

Thermal cycling conditions were as follows: 95 °C for 5 min; followed by 40 cycles of 95 °C for 5 s, 58 °C for 45 s, and 72 °C for 30 s; followed by melt curve analysis from 65 °C to 95 °C in 0.5 °C increments, with 5 s per step, plus plate reading. Each reaction was performed in triplicate. Threshold cycle (Ct) values were used for further analysis. Relative gene expression was calculated using the 2^−ΔΔCt^ method, normalized to 16S–23S rDNA expression [28].

All experiments were performed in triplicate. Genes with a *p*-value < 0.05 and a fold-change ≥ 1.5 were considered significantly differentially expressed. 

### 2.9. Membrane Integrity Assessment by Propidium Iodide Staining

Fifty microliters of overnight cultures of *V. parahaemolyticus* (WT, VP: △*tdh2*, and C-△*tdh2*) were transferred into 5 mL of LBNB and cultured for 12 h at 37 °C with shaking at 225 rpm. Then, 100 μL of the culture was transferred into 5 mL of LBNB containing 0.9% bile and incubated for 2 h at 37 °C. After incubation, 1 mL of the culture was centrifuged at 3000 rpm for 5 min. The supernatant was removed, and the cell pellet was collected. The cells were washed with PBS and resuspended in 200 μL of PBS (10^8^ CFU/mL). Then, 10 μL of propidium iodide (PI) (1 mg/mL in ddH_2_O) was added to the suspension, mixed thoroughly, and incubated at 37 °C for 20 min in the dark. The stained suspension was centrifuged at 3000 rpm for 5 min. The pellet was washed twice with PBS and resuspended in PBS. Finally, the stained cells were mounted on a coverslip and observed using a TCS SP5 II laser confocal microscope (Leica, Düsseldorf, Germany).

### 2.10. Erythrocyte Hemolysis Assay

For the erythrocyte hemolysis test, rabbit red blood cells were diluted in phenolsulfonphthalein-free Dulbecco’s Modified Eagle Medium (DMEM; BOSTER, Wuhan, China) to a final concentration of 5% (RBC-DMEM). *V. parahaemolyticus* RIMD2210633 WT and VP-∆*tdh2* were cultured overnight and adjusted to an OD_600_ of 0.4. A 10 μL aliquot of each culture was added to 500 μL of RBC-DMEM and incubated at 37 °C for 1 to 6 h without shaking. Every 10 min, the mixtures were gently resuspended to release hemoglobin. At hourly intervals, the samples were centrifuged at 12,000 rpm for 1 min, and 200 μL of the supernatant was transferred to a 96-well plate to measure absorbance at 540 nm using an Infinite 200 multifunction microplate reader (Tecan, Männedorf, Switzerland). As controls, red blood cells lysed with 2% Triton X-100 served as the positive control, and DMEM containing 5% erythrocytes without *V. parahaemolyticus* served as the negative control.

### 2.11. LC-MS Analysis on Intracellular Metabolites of V. parahaemolyticus

*V. parahaemolyticus* WT and VP: ∆*tdh2* were cultured for 12 h at 37 °C and centrifuged at 10,000 rpm for 5 min. The resulting pellet was prepared for LC-MS analysis. Sixty milligrams of bacterial cells were transferred to a 2 mL centrifuge tube, followed by the addition of 500 μL methanol (−20 °C) and 500 μL ddH_2_O (4 °C). The mixture was vortexed for 30 s. Next, 100 mg of glass beads were added, and the mixture was vortexed for 1 min, then subjected to three freeze–thaw cycles. Afterward, it was centrifuged at 13,000 rpm for 10 min at 4 °C. The supernatant was concentrated at 1500 g for 1–2 h at 35 °C using a vacuum centrifuge (SPD210-115 SpeedVac, Thermo Fisher Scientific Inc., USA), then resuspended in 300 μL of methanol/water (1:1, *v/v*, 4 °C) and filtered through a 0.22 μm membrane for further analysis.

Metabolomic analysis was performed using an Agilent 1290 UHPLC-6540 QTOF system equipped with an electrospray ionization (ESI) source operating in both positive (ESI^+^) and negative (ESI^−^) ion modes. Metabolic profiles were acquired in the *m/z* range of 50–1000. Separation was achieved using a BEH C18 column (1.7 μm, 2.1 × 100 mm; Waters, Milford, MA, USA). The mobile phases were 0.1% formic acid in water (A) and acetonitrile (B). The gradient elution conditions were as follows: 60% B from 0 to 6 min; increase to 98% B from 6 to 12 min, held at 98% from 12 to 13 min; decreased to 2% B from 13 to 14 min, and held for 1 min. The flow rate was 0.4 mL/min, column temperature was maintained at 40 °C, and the injection volume was 2 μL. Instrument parameters were as follows: capillary voltage, 4000 V; needle voltage, 1000 V; sheath gas temperature, 250 °C; atomizer pressure, 45 psi; dry gas flow rate, 10 L/min; drying gas temperature, 350 °C; and fragmentation voltage, 140 V.

### 2.12. Statistical Analysis

Data of LC-MS was analyzed by Simca-P 13.0. Potential differential metabolites were selected according to variable importance on projection (VIP > 1), loading plot, and critical *p*-values from Student’s *t*-test (*p* < 0.05) using SPSS 19.0. For the identification of metabolites, METLIN (https://metlin.scripps.edu/auth-login.html, accessed on 30 December 2022) and KEGG (https://www.kegg.jp/, accessed on 30 December 2022) were used. All experiments were repeated three times and the data were obtained at least in triplicate. The data were analyzed by performing a *t*-test or ANOVA using SPSS 19.0 at a significance level of *p* < 0.05. All data are presented as mean ± SD.

## 3. Results

### 3.1. Expression of tdh2 Significantly Increased as Response to Environmental Stress

To examine the relationship between *tdh2* expression and environmental conditions, *V. parahaemolyticus* WT was subjected to cold shock (4 °C) or bile treatment (final concentration: 0.9%), and *tdh2* expression was quantified by RT-qPCR. A marked increase in *tdh2* expression was observed under both conditions (Table 4). Specifically, *tdh2* expression increased approximately 1.32 × 10^4^-fold in response to bile and 3.65 × 10^2^-fold in response to cold shock, independent of total colony counts.

### 3.2. Construction and Validation of the tdh2 Knockout and Complemented Strains

**Figure 1:**
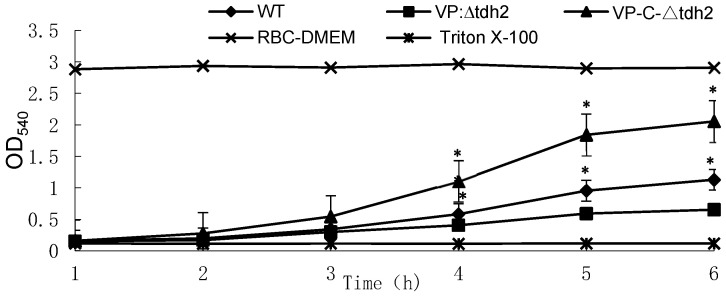
Changes in hemolytic activities of *V. parahaemolyticus* WT, VP: ∆*tdh2*, and VP-C-∆*tdh2* in RBC-DMEM containing 5% of red blood cells of rabbit at OD_540_. Red blood cells of rabbit lysed with 2% Triton X-100 and RBC-DMEM were used as the positive and negative control, respectively. The data were obtained in triplicate. “*“ indicates a significant difference (*p* < 0.05).

Except for horse erythrocytes, TDH can lyse erythrocytes from humans, sheep, and rabbits, resulting in β-hemolysis. We knocked out the *tdh2* gene in the WT strain and constructed a complemented strain, VP-C-∆*tdh2*, to examine the resulting changes in hemolytic activity among the three strains. Differences in hemolytic activity appeared after 3 h. VP-C-∆*tdh2*, which carried the low-copy plasmid pACYC184 containing the *tdh2* expression cassette, showed the highest hemolytic activity (Figure 1). The hemolytic activity of VP: ∆*tdh2* was significantly lower than that of the WT due to the absence of *tdh2*, but complementation restored the activity (*p* < 0.01) (Figure 1). The growth curves and viable cell counts of the three strains showed no significant differences, indicating that hemolytic activity mainly depended on *tdh2* rather than bacterial abundance.

### 3.3. tdh2 Improved the Tolerance of V. parahaemolyticus to Acid Stress

**Figure 2:**
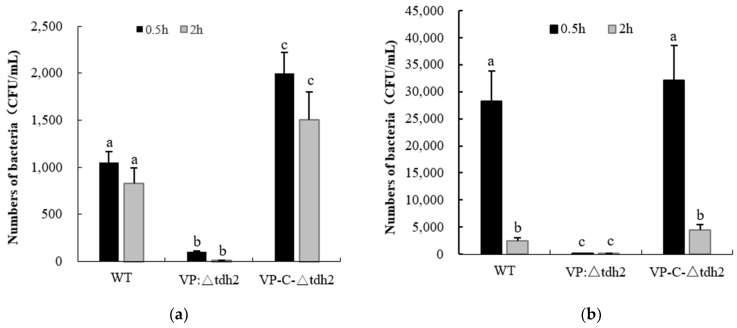
Survival numbers of *V. parahaemolyticus* WT, VP: △*tdh2*, and C-△*tdh2* in LBNB with pH 3 (**a**) and pH 4 (**b**) at 37 °C for 0.5 h and 2 h. The data were obtained at least in triplicate. There is a significant difference (*p* < 0.05) between numerical values marked with different superscripts (a, b, c).

As an acid-intolerant bacterium, *V. parahaemolyticus* is only marginally viable at pH 5, considered a sublethal condition, though high salt concentrations can aid its survival via the expression of *cadA* (VP2890) [23]. We examined the survival of the WT, VP: ∆*tdh2*, and VP-C-∆*tdh2* strains (final concentration: ~2 × 10^6^ CFU/mL) in LBNB adjusted to pH 3 and pH 4. Significantly higher survival was observed in the WT and VP-C-∆*tdh2*. After 2 h at pH 3, no viable VP: ∆*tdh2* cells were detected, while the WT and VP-C-∆*tdh2* retained 0.041% and 0.075% survival, respectively (Figure 2a). A similar trend was observed at pH 4. After 0.5 h of incubation, the survival rates were 1.41% for the WT, 0.01% for VP: ∆*tdh2*, and 1.61% for VP-C-∆*tdh2*. At 2 h, survival rates were 0.13% and 0.23% for the WT and VP-C-∆*tdh2*, respectively, with almost no surviving cells detected for VP: ∆*tdh2* (Figure 2b). These findings suggest that *tdh2* deletion increases acid susceptibility, while its overexpression enhances acid tolerance.

### 3.4. tdh2 Improved the Bile Tolerance of V. parahaemolyticus and Membrane Integrity

**Figure 3:**
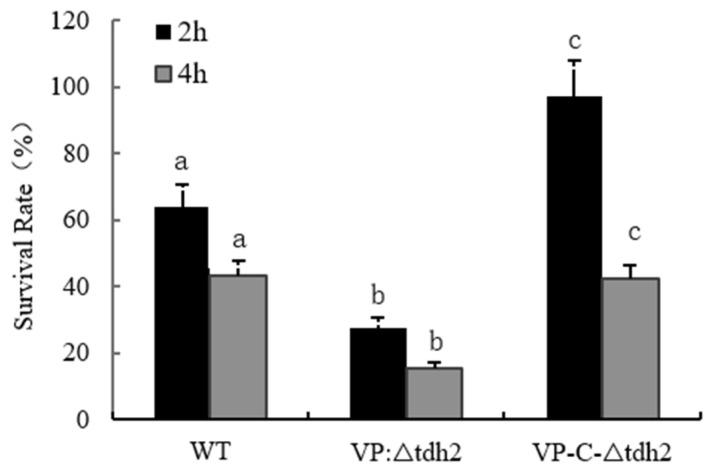
Survival rates of *V. parahaemolyticus* WT, VP: △*tdh2*, and C-△*tdh2* in LBNB with 2% of bile salt at 37 °C for 2 h and 4 h. The survival rate was determined as “colonies on the plate/the number of inoculation × 100%”. Data were obtained at least in triplicate. There is a significant difference (*p* < 0.05) between numerical values marked with different letters.

Bile, an osmotic stressor, is one of the key inducers and critical factors for intestinal colonization by *V. parahaemolyticus*. At least 77 genes, including the *tdh2* and *Vp-PAI* genes, are induced by bile during infection [29,30]. We assessed the survival of WT, VP: ∆*tdh2*, and VP-C-∆*tdh2* under 2% bile stress. The VP: ∆*tdh2* strain showed significantly reduced bile tolerance compared to the WT and VP-C-∆*tdh2*. After 2 h of bile exposure, the survival rates were 63.7% (WT), 27.48% (VP: ∆*tdh2*), and 97.3% (VP-C-∆*tdh2*). After 4 h, survival decreased to 15.4% for VP: ∆*tdh2*, while the WT and VP-C-∆*tdh2* maintained 43.4% and 42.3% viability, respectively (*p* < 0.05) (Figure 3).

**Figure 4:**
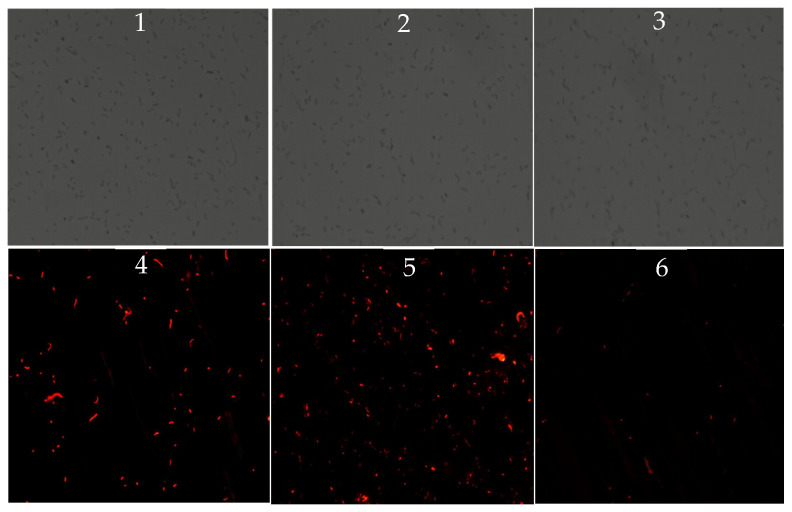
Inverted microscope observations of *V. parahaemolyticus* treated with 0.9% of bile for 2 h at 37 °C (**1**–**3**: observations of WT, VP: △*tdh2*, and VP-C-△*tdh2* under white light; **4**–**6**: observations of WT, VP: △*tdh2*, and VP-C-△*tdh2* under fluorescent light). The scale bar is 50 μm. The samples were mixed with 10 μL of PI (1 mg/mL) at 37 °C for 20 min without light.

PI, a nuclear stain that can only penetrate damaged membranes, was used to assess membrane integrity following bile exposure. While the total number of cells (under white light) was similar among all three strains, fluorescence intensity (indicative of membrane damage) was highest in VP: ∆*tdh2*, followed by the WT. Almost no red fluorescence was observed in VP-C-∆*tdh2* (Figure 4). These results indicate that *tdh2* plays a role in protecting or restoring membrane integrity under bile stress, though the precise mechanism remains unclear.

### 3.5. Gene Expression Changes in tdh2 Deletion Strain, Focusing on ABC Transporters

**Figure 5:**
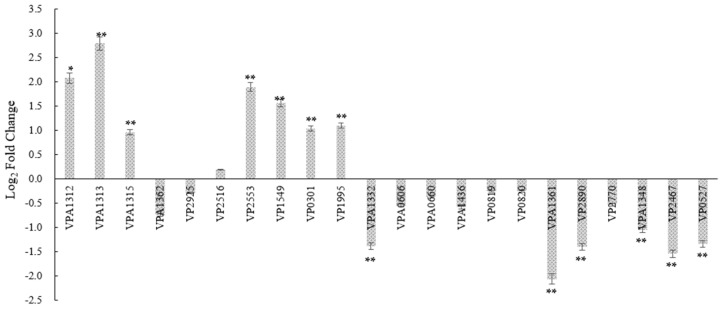
The log_2_-fold change of the relative expression levels of 22 genes in *V. parahaemolyticus* VP: △*tdh2* compared with the WT at 37 °C for 12h in LBNB. The relative mRNA expression levels of each gene normalized to 16s–23s rDNA expression were calculated by using the 2^−ΔΔCt^ method. Genes with a p-value < 0.05 and a differential expression fold change ≥ 1.5 (log_2_-fold change ≥ 0.585) are significantly differentially expressed. “*” and “**” indicate significant differences from *V. parahaemolyticus* WT at *p* < 0.05 and *p* < 0.01. The data were obtained at least in triplicate. All data are presented as mean ± SD.

To investigate the relationship between *tdh2* and stress protection, transcriptional analysis of 22 genes was performed in *V. parahaemolyticus* WT and VP: △*tdh2*. These 22 genes included major *tdh2* regulators—VPA1332 (*vtrA*), VPA1348 (*vtrB*), VP0819 (*toxS*), and VP0820 (*toxR*)—as well as the outer membrane proteins VP2516 (*opaR*) and VP2467 (*ompU*), efflux-related ABC transporters (VP0301, VP1995, VPA0606, and VPA1436), the Na^+^/H^+^ antiporter regulatory protein VP0527 (*nhaR*), stress-response factors for bile and acid such as VP2553 (*rpoS*) and VP2890 (*cadA*), genes located upstream and downstream of *tdh2* (VPA1312, VPA1313, and VPA1315), and other functional genes, including VPA1362 (*ESPD*), VP2925 (*rplA*), VP1549 (*orf10*), VPA1361 (T3SS2 effector protein), and VP2770 (*tuf*). Compared to the WT, seven of the 22 genes—VPA1312, VPA1313, VPA1315, VP2553, VP1549, VP0301, and VP1995—were significantly upregulated in VP: △*tdh2* by 4.23-, 6.94-, 1.95-, 3.72-, 2.96-, 2.06-, and 2.14-fold, respectively. Meanwhile, six genes—VPA1332 (*vtrA*), VPA1361, VP2890, VPA1348 (*vtrB*), VP2467, and VP0527—were significantly downregulated by 2.6-, 4.17-, 2.64-, 2.1-, 2.90-, and 2.53-fold, respectively (*p* < 0.05; fold change > 1.5) (Figure 5). However, the expression of *toxS* (VP0819) and *toxR* (VP0820) did not significantly change, even though *vtrA* and *vtrB* were downregulated.

**Figure 6:**
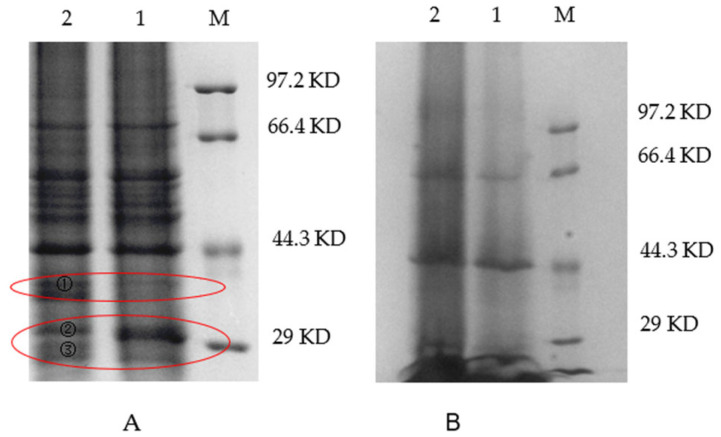
SDS-PAGE electrophoresis of the cells (**A**) and supernatant (**B**) of *V. parahaemolyticus* in LBNB at 37 °C for 18 h (M: protein makers; 1: WT; 2: VP: △*tdh2*). Band 1, 2, and 3 on land 2 in red circle were different bands compared with WT. Band 3 was cut off for LC-MS analysis.

**Figure 7:**
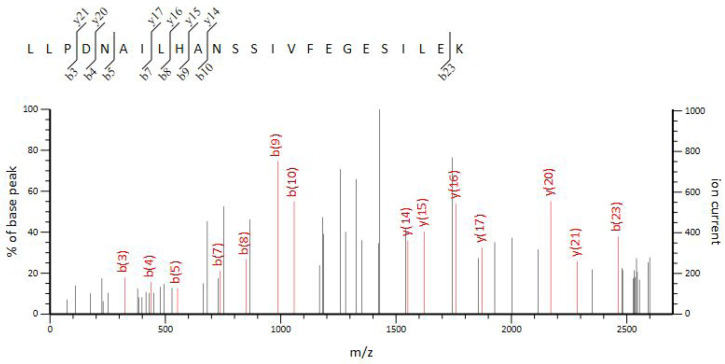
LC-MS analysis of band 3.

Correspondingly, differences in protein expression between the WT and VP: △*tdh2* were examined via SDS-PAGE for both cellular and secreted proteins. The supernatant protein profiles of VP: △*tdh2* and the WT were nearly identical, but the cellular protein patterns differed. Three protein bands, with molecular weights between 29 kDa and 44.3 kDa, showed changes in VP: △*tdh2* (Figure 6A). These were labeled as bands 1, 2, and 3. Band 1 showed increased expression, band 2 showed decreased expression, and the ~29 kDa band 3 was absent in the WT. LC-MS analysis suggested that band 3 was likely an ABC transporter ATP-binding protein (Figure 7), while bands 1 and 2 were predicted hypothetical proteins. This result aligned with the RT-qPCR findings, which showed significantly upregulated expression of two ABC transporters—VP0301 and VP1995—in VP: △*tdh2*. The elevated expression of these ABC transporters, which are located on the cell membrane of *V. parahaemolyticus*, might enhance the efflux of harmful substances, providing increased resistance to acid and bile stress in the absence of *tdh2*.

### 3.6. Altered Membrane Phospholipid Composition and Metabolism in tdh2 Mutant

**Figure 8:**
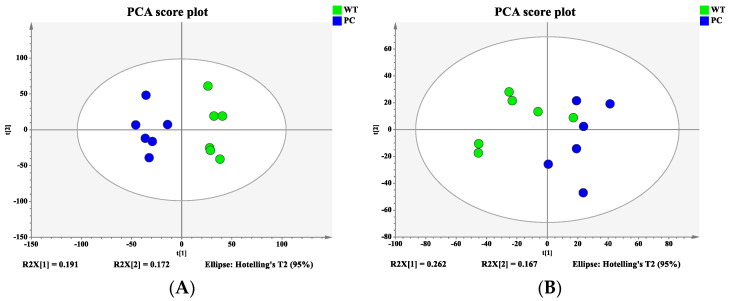
Principal component analysis (PCA) score plot: (**A**) ESI^+^; (**B**) ESI^−^.

**Figure 9:**
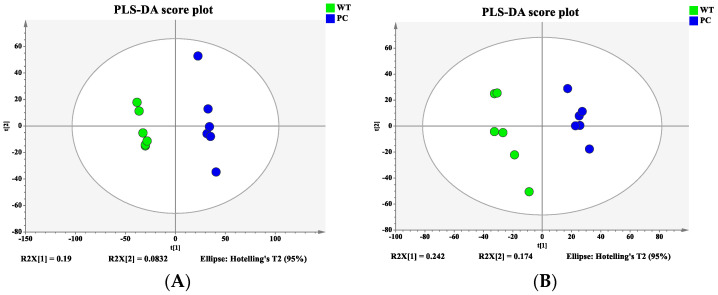
Partial least squares discriminant analysis (PLS-DA) score plot: (**A**) ESI^+^; (**B**) ESI^−^.

Bacterial samples from the WT and VP: △*tdh2* were subjected to metabolomic profiling using the 1290UHPLC-6540QTOF system in both ESI^+^ and ESI^−^ modes. PCA and PLS-DA models were constructed to visualize the metabolic differences between the two strains. Good separation was observed in ESI^+^ (Figure 8A), while partial overlap occurred in ESI− (Figure 8B). To further distinguish the metabolic differences between the WT and VP: △*tdh2*, the PLS-DA models were applied for multivariate statistical analysis. Clear discrimination was achieved with the following model parameters: R^2^X = 27.3%, R^2^Y = 99.8%, and Q^2^ = 69.7% in ESI^+^ (Figure 9A); and R^2^X = 41.7%, R^2^Y = 98.8%, and Q^2^ = 88.5% in ESI− (Figure 9B). The two groups displayed distinct clustering, confirming significant differences in their metabolic profiles.

The detected ions were filtered using VIP values (VIP > 1) and P-values (P < 0.05), and selected metabolites were identified by MS/MS spectra and the METLIN database. Twelve differential metabolites between the WT and VP: △*tdh2* were identified (Table 5). Notably, glycerophospholipids, including phosphatidylserine (PS) and phosphatidylglycerol (PG), were upregulated in VP: △*tdh2* compared to the WT. In contrast, nucleotides and cofactors such as guanosine and its derivatives, adenine, uridine, thymine, and flavin mononucleotide (FMN), were downregulated. These results indicate that *tdh2* deletion affects membrane composition and may impair basic metabolic activities in *V. parahaemolyticus*.

## 4. Discussion

Evidence shows that nearly 95–99% of *V. parahaemolyticus* clinical isolates from food poisoning patients are KP^+^ and *tdh*^+^, while the strains isolated from the environment or food are often *tdh*-negative. Several factors may contribute to the selection of pathogenic strains in the human intestine, independent of their low prevalence in the environment. A total of 565 genes has been identified as potentially essential for the growth and viability of pandemic *V. parahaemolyticus* in vitro, including virulence factors, structural components, transcriptional regulators, and mediators of biological processes linked to intestinal colonization [27]. However, although *tdh2* is one of the most important virulence genes and is highly expressed under adverse environmental conditions, its role in the tolerance of *V. parahaemolyticus* to environmental stress remains unclear beyond its known pore-forming function [19,20]. In this study, we investigated the possible role and mechanism by which *tdh2* contributes to environmental stress tolerance in *V. parahaemolyticus*.

Our results showed that *tdh2* expression increased by approximately 1.32 × 10^4^-fold and 3.65 × 10^2^-fold after 4 h of exposure to bile and cold shock, respectively, suggesting a potential role in stress protection. Therefore, we constructed a *tdh2*-deletion mutant (VP: △*tdh2*) and a complementary strain (VP-C-△*tdh2*) to evaluate the effects of *tdh2* under simulated gastrointestinal conditions. Gastric acid and bile are among the most significant environmental stressors affecting intestinal colonization. Our findings demonstrated that the survival of the WT was significantly higher than that of VP: △*tdh2* but lower than that of VP-C-△*tdh2* under acid and bile stress, indicating a protective function of *tdh2*. As an acid-intolerant bacterium, *V. parahaemolyticus* is highly sensitive to low pH values. In our study, VP: △*tdh2* showed much lower survival than the WT and VP-C-△*tdh2* at pH 3 and 4. This sensitivity may be related to VP2890 (*cadA*), which encodes lysine decarboxylase [23]. *cadA* is the only known inducible system and acid-sensing signaling factor that can directly bind acid and regulate *rpoS* expression in *V. parahaemolyticus*, regardless of salt concentration [23]. In this study, *cadA* expression in VP: △*tdh2* was downregulated 2.64-fold compared to the WT, possibly contributing to reduced acid resistance and lower bacterial survival. *toxR* may act as a connecting regulator between *tdh2* and *cadA* via co-regulation of both genes.

Bile plays a dual role in *V. parahaemolyticus*, acting both as an antimicrobial agent and as an inducer of gene expression, including *tdh2* and T3SS2-related genes regulated by *vtrA* [31]. In the human intestine, high levels of TDH expression promote the growth of *V. parahaemolyticus* by enabling Fe^2+^ acquisition through erythrocyte lysis via pore formation in the cell membrane. In our study, *tdh2* improved the survival of *V. parahaemolyticus* under bile stress, even in the absence of erythrocytes. This protective effect may be linked to 13 genes, including seven upregulated and six downregulated in the VP: △*tdh2* strain. Among them, VPA1332 (*vtrA*), VPA1348 (*vtrB*), VP2467 (*ompU*), VP0301 and VP1995 (ABC transporters), VP0527 (*nhaR*), and VP2553 (*rpoS*) were particularly important (Figure 5).

The direct regulatory relationships among bile, *vtrA*, *vtrB*, and *tdh2* have been previously documented. *V. parahaemolyticus* detects bile salts via a 1:1 VtrA–VtrC complex, which binds bile acids [32]. VtrA then binds to the 284 bp promoter region of *vtrB*, initiating its expression. In turn, VtrB activates the promoters of *tdh2* and *Vp-PAI*, inducing expression of *tdh2* and T3SS2-related genes [32]. Both *vtrA* and *vtrB*, as master regulators of *tdh2*, function similarly to *toxRS* in adapting to bile, acid stress, and antimicrobial exposure and are essential for bile-induced *tdh2* expression. In this study, *vtrA* and *vtrB* expression in VP: △*tdh2* was downregulated by 2.6-fold and 2.1-fold, respectively, indicating a reduced ability of this mutant to sense bile. *toxRS* is another important regulatory system for *tdh2*, and its activation is required for *vtrB* induction [33]. It also regulates the expression of outer membrane proteins OmpU and OmpN [27]. A *toxRS*-deficient strain shows impaired colonization ability, which can be restored by *ompU* complementation [34]. However, in our study, the expression of VP0819 (*toxS*) and VP0820 (*toxR*) was not significantly affected by *tdh2* deletion, further highlighting the dominant regulatory role of *vtrA* and *vtrB* over *tdh2*.

Bile is the substrate of OmpU and TolC (VP1998). OmpU also acts as a signal to activate *rpoE*, an alternative sigma factor important for the cell envelope stress response and intestinal colonization of *V. parahaemolyticus* [35]. In our results, *ompU* was downregulated 2.90-fold in VP: △*tdh2*, likely as a stress response to bile pressure, in order to prevent bile transport. Many efflux systems in *V. parahaemolyticus* protect the bacterium from the toxic effects of bile, including ABC transporters, resistance/nodulation/cell division (RND) transporters, toxic compound extrusion (MATE) family members, the major facilitator superfamily (MFS), and small multidrug resistance (SMR) proteins [36]. RND transporters such as VmeAB, VmeCD, and VmeTUV have been reported to be mainly involved in bile acid resistance [37]. In our study, the upregulation of two ABC transporters, VP0301 and VP1995, reflected the response of the damaged cell membrane to toxin invasion and the effort to efflux bile. The PI staining results (Figure 4) showed that *tdh2* could protect the bacteria from bile-induced damage by maintaining cell membrane integrity, which may be related to *ompU* and ABC transporters. ABC transporters typically lack DNA-binding domains and rely on other regulatory proteins to transmit signals. They may indirectly regulate *tdh2* via the quorum sensing system or a novel two-component system. The regulatory relationship between ABC transporters and *tdh2* requires further study.

VP0527 (*nhaR*) is a Na^+^/H^+^ antiporter regulatory protein that transcriptionally activates the expression of *nhaA* and *osmC* [38]. The downregulation of VP0527 (*nhaR*) in VP: △*tdh2* may consequently cause decreased expression of *nhaA* and disrupt the transmembrane electrochemical potential of H^+^. An increased intracellular H^+^ concentration leads to bacterial death due to reduced adaptability to acidification. Moreover, the imbalance of Na^+^ and H^+^ causes a decrease in membrane potential, resulting in the depolarization of the cell membrane and increased permeability.

Modulation of *rpoS* expression is another important mechanism during exposure to environmental stress; it activates genes necessary for survival under stress and plays a significant role in *V. parahaemolyticus* adaptation to bile and acid [23,39]. VP2553 (*rpoS*) was upregulated 3.72-fold in VP: △*tdh2*, indicating stress-induced stimulation for survival under adverse conditions. Currently, there is no evidence of a direct regulatory relationship between *ompU*, *rpoS*, and *tdh2*. However, the literature suggests that OmpU is directly regulated by ToxR, which subsequently activates *rpoS* expression. After *tdh2* deletion, changes in *ompU* and *rpoS* expression may be related to ToxR, which can directly bind to the promoter region of *tdh2*.

**Figure 10:**
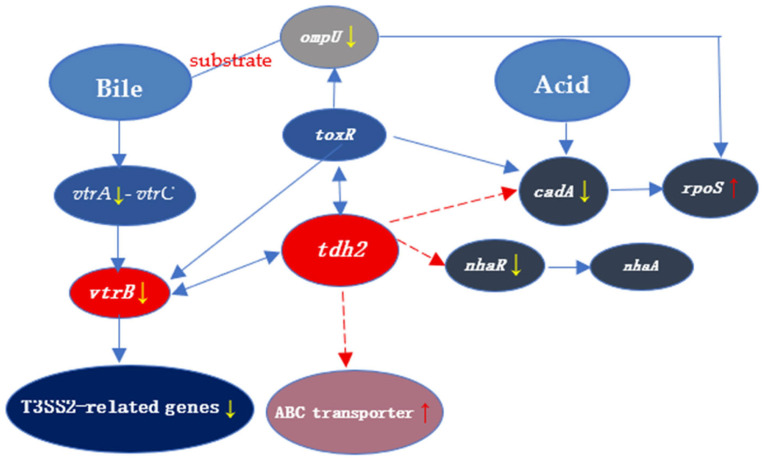
The transcriptional relationship between *tdh2* and the 8 genes involved in our study. The red and yellow arrows indicate upregulation and downregulation of the expression, respectively. The dashed and solid lines represent unknown and well-studied regulation, respectively. *V. parahaemolyticus* senses bile salts by a 1:1 complex formed by VtrA and VtrC. Bile acids such as TDC and CDC can bind to the hydrophobic pocket in the VtrA-VtrC complex. Then, VtrA binds to the 284 bp promoter region of *vtrB*, which in turn binds to promoters of *tdh2* and Vp-PAI to induce the expression of *tdh2* and T3SS2-related genes. There is currently no evidence for the direct regulatory relationship between genes (*ompU* and *rpoS*) and *tdh2*. OmpU is regulated by *toxR* directly and then activates the *rpoS* expression. After the deletion of *tdh2*, the expression changes of o*mpU* and *rpoS* may be related to *toxR*, which can directly bind to the promoter region of *tdh2*. VP2890 (*cadA*) is an acid-sensing signaling factor that can directly bind to acids, as well as regulate the *rpoS* expression. ABC transporters may indirectly regulate *tdh2* by the quorum sensing system or a new two-component system. The regulatory relationship between ABC transporters and *tdh2* needs further study.

The relationships between *tdh2* and these genes are summarized in Figure 10.

The outer membrane of *V. parahaemolyticus* is rich in phospholipids such as phosphatidylethanolamine (PE), phosphatidylglycerol (PG), and cardiolipin (CL). Among the 12 differential metabolites identified via LC-MS/MS (Table 5), PS (17:2 (9Z,12Z) /0:0), PS (20:1 (11Z) /0:0), and PG (17:0/0:0) participate in glycerophospholipid metabolism and serve as metabolic intermediates of phospholipids involved in cell membrane recognition and signal transduction. Glycerophospholipids contribute to forming a protective barrier and modulating membrane traffic [40]. The increased release of glycerophospholipids in VP: △*tdh2* indicates damaged cell membrane integrity. Following *tdh2* deletion, the downregulation of VP0527 (*nhaR*) reduced membrane potential due to Na^+^/H^+^ imbalance, leading to membrane depolarization, damage, and increased permeability. Therefore, *tdh2* not only functions as an exotoxin but also regulates cell membrane stability by affecting ion balance (e.g., Ca^2+^, K^+^). PS and PG are negatively charged, maintaining a negative charge on the cell membrane surface; they also serve as binding sites for cations such as Ca^2+^ and Mg^2+^ and influence membrane protein activity. When the cell membrane is damaged, bacteria upregulate PG synthesis to repair membrane structure, explaining the increased PG content in VP: △*tdh2*. PS or its derivatives may accumulate temporarily to enhance membrane stability under stress. These metabolic changes are caused by a series of regulatory networks related to *tdh2* deletion.

Our LC-MS/MS results showed that the contents of adenine, guanosine, hypoxanthine, and uridine decreased significantly in VP: △*tdh2*. Leakage of the proton gradient (ΔpH) leads to decreased oxidative phosphorylation efficiency, which affects adenosine triphosphate (ATP) synthesis and ultimately inhibits nucleotide production. Due to membrane damage, *V. parahaemolyticus* prioritizes resources for membrane repair by enhancing phospholipid PG and PS synthesis, thereby reducing the expression of genes related to nucleotide synthesis (e.g., *pur* and *pyr*).

Oxidative phosphorylation is the metabolic pathway in which cells generate ATP by using energy released from nutrient oxidation (such as glucose) through the electron transport chain (ETC). In *V. parahaemolyticus*, this occurs at the plasma membrane. The ETC is a series of protein complexes and electron carriers, including Complexes I, II, ubiquinone, III, cytochrome c, and Complex IV. Complexes I, III, and IV use energy from electron transfer to pump H^+^ into the intermembrane space, creating an electrochemical gradient known as the proton motive force (PMF). The H^+^ gradient then drives protons back through ATP synthase to produce ATP. Without a functioning ETC, oxidative phosphorylation cannot occur, severely limiting cellular energy production.

Flavin mononucleotide (FMN) is a cofactor of NADH dehydrogenase, the “entry enzyme” for oxidative phosphorylation, primarily present in Complex I [41]. As an electron carrier, FMN accepts 2 H^+^ from NADH and is reduced to FMNH_2_. Subsequently, FMNH_2_ transfers electrons to Fe-S clusters and ultimately to ubiquinone [41,42]. By participating in proton pumping, Complex I utilizes the energy released through FMN-mediated electron transfer to pump 4 H^+^ from the cytoplasm to the periplasmic space, forming a proton gradient. A decrease in intracellular FMN leads to Complex I dysfunction, preventing effective electron transfer from NADH. This results in reduced ETC efficiency → decreased proton pumping → weakened proton gradient → diminished ATP synthesis.

PMF is the electrochemical gradient of protons (H^+^) across the inner membrane generated by the ETC. It comprises the chemical gradient ΔpH (difference in proton concentration, ~30%) and the electrical gradient ΔΨ (transmembrane potential, charge difference across the membrane, ~70%). PMF provides energy for ATP synthase to phosphorylate ADP to ATP. The transmembrane potential ΔΨ, caused solely by charge separation, is the primary driving force for ATP synthesis and is essential for maintaining membrane integrity. If membrane permeability increases and the transmembrane potential dissipates, H^+^ can reflux freely without passing through ATP synthase, blocking ETC electron transfer and preventing ATP synthesis.

The decreases in FMN (LC-MS/MS) and *nhaR* expression (RT-qPCR) observed in our results led to reduced ETC efficiency → decreased proton pumping → weakened proton gradient → diminished ATP synthesis. The elevated expression of ABC transporters observed typically alters membrane permeability to specific substances through active efflux or uptake, as confirmed by PI staining.

**Figure 11:**
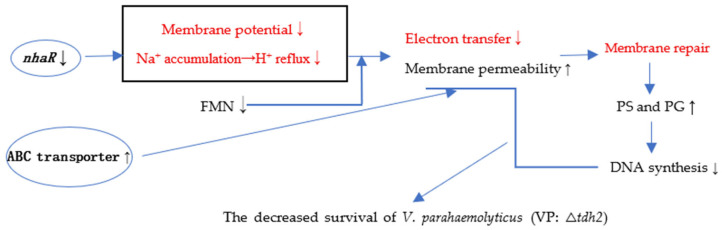
The speculation about the decreased survival of *V. parahaemolyticus* (VP: △*tdh2*). The red and black words indicate speculation and confirmed results in this study, respectively. ↑ and ↓ represent upward and downward, respectively.

In summary, the deletion of *tdh2* resulted in decreased *nhaR* expression and increased expression of the ABC transporters VP0301 and VP1995. The decreased *nhaR* expression caused accumulation of Na^+^ in cells and blocked H^+^ reflux. Consequently, altered *nhaR* and ABC transporter activity led to increased membrane permeability in *V. parahaemolyticus* cells and decreases in PMF and FMN. Electron transfer was blocked, resulting in reduced ATP and DNA synthesis. This explains why VP: △*tdh2* cells were more prone to death. To compensate, the expression of bile-sensing genes (*vtrA* and *vtrB*) and the bile-transporting gene (*ompU*) in VP: △*tdh2* decreased. Simultaneously, the cells actively synthesized phospholipids to repair the membrane, leading to increased levels of the phospholipid-related intermediate metabolites PG and PS. The proposed mechanism for the decreased survival of *V. parahaemolyticus* VP: △*tdh2* is illustrated in Figure 11.

## 5. Conclusions

Serious food poisoning caused by *V. parahaemolyticus* occurs worldwide every year. A decrease in the survival of *tdh^+^* strains in the human intestine is a key factor in preventing such incidents. The reason why strains harboring *tdh2* exhibit better competitive colonization compared to non-pathogenic strains remains unclear. Our results demonstrated that *tdh2* confers protection to *V. parahaemolyticus* against gastrointestinal stresses, particularly acid and bile stress, resulting in increased survival compared to the *tdh^-^* strains. *tdh2* helps maintain cell membrane permeability through the regulation of *ompU* and ABC transporters and sustains electron transfer efficiency via the regulation of *nhaR*. Increased membrane permeability, decreased electron transfer efficiency, and the consequent reduction in ATP, DNA, and RNA synthesis were responsible for the higher mortality rate observed in *V. parahaemolyticus* VP: △*tdh2*.

The deletion of *tdh2* caused changes in the expression of multiple genes. Except for *vtrA* and *vtrB*, the regulatory relationships between *tdh2* and other genes, such as ABC transporters, remain unknown. ABC transporters may interact with *tdh2* through environmental pressure, the quorum sensing system, or novel two-component systems. Therefore, further research—including bioinformatic analyses, promoter binding assays, and protein–protein interaction studies—is needed to better elucidate the regulatory network associated with *tdh2*. Moreover, this study proposes a novel protective role of *tdh2* that is distinct from its known pore-forming activity and toxicity. Understanding this new role will help us more effectively prevent and control outbreaks of foodborne diseases caused by *V. parahaemolyticus*.

## Figures and Tables

**Table 1:** Strains and plasmids used in this study.

Strains and Plasmids	Characteristics	Sources
*V. Parahaemolyticus* RIMD2210633	*tdh1*^+^, *tdh2*^+^, O3:K6 serotype	Provided by Prof. Zhou Dongsheng
VP: ∆*tdh2*	*tdh1*^+^, *tdh2*^−^, O3:K6 serotype	Constructed in this study
VP-C-△*tdh2*	*tdh1*^+^, *tdh2*^+^, O3:K6 serotype	Constructed in this study
*E. coli* S17-1-pir	RP4-2, *pir*	Provided by Prof. Zhou Dongsheng
*E. coli* DH5α	F^−^, *SupE 44*, *φ80dlacZ*△*M15*, △*(lacZYA-argF) U169*, *hsdR17* (rk^−^, mk**^+^**), *recA1*, *endA1*, *gyrA96*, *thi-1*, *relA1*	Kept in our lab
pDS132	Cm^R^, *sacB*, mob RP4, Suicide plasmid	Provided by Prof. Zhou Dongsheng
pDS132-*tdh2*	Cm^R^, harboring truncated *tdh2* region	Constructed in this study
pACYC184	Cm^R^, Tc^R^	Purchased from ATCC (Rockville, MD, USA)
pACYC184-*tdh2*	Cm^R^, *tdh2*^+^, Tc^R^	Constructed in this study

**Table 2:** Primers used for mutant construction.

Primers	Sequences
1314-A	AAACTGCAGTGACGTAGAGTCTAATCCAT
1314-B	AAAAAAACCTCTGAATTGATTAATAACTTTGCCAG
1314-C	TCAGAGGTTTTTTTCCAATGCAAAACTAGA
1314-D	ACTGCATGCATATTTCCTCATCGTAA
1314-E	AAGGTTATTTCTTTCCCCTAGCATC
1314-d	TTTCGGCATGAGACTAGGGGAAT
*tdh2*-F	CTTTTAATACCAATGCACC
*tdh2*-R	GTTGAAGCTGTACTTGATCTG
*sacB*-F	ACGGCACTGTCGCAAACTAT
*sacB*-R	TTCCGTCACCGTCAAAGAT
*tdh*-HB-F	CGCGGATCCATCTACCAAGCGATAAGGC
*tdh*-HB-R	CCCAAGCTTGAAGCGAATAAATAGCGTG
pDS132-F	GGCAGGTATATGTGATGGGT
pDS132-R	GGATGTAACGCACTGAGAAG

**Table 3:** Primers used in RT-qPCR.

Primers	Sequences	Amplicon Size (bp)
16-23S-F	GCTGACAAAACAACAATTTATTGTT	170
16-23S-R	GGAGTTTCGAGTTGATGAAC	
VP1312-F	ATACTAAGATTATGCCGTCCTG	127
VP1312-R	TTCGCCGAGATTGTTTGC	
VP1313-F	GGCTTTGTTGCGTAATAGTGA	86
VP1313-R	AATGCCTTATCGCTTGGTAG	
VP1315-F	TCGCTTCTGATGGTTACACTT	133
VP1315-R	TAATGCCTTATCGCTTGGTC	
VP2553-F	GCATTTTGCTGACATCTTCG	149
VP2553-R	CACCATTCGCTTGCCTATT	
VPA1332-F	TGCTCCTCGCCTTGTGTG	130
VPA1332-R	AAATGGGCTCTGATGTTACG	
VPA1348-F	GAGAGAAACGCAGACGAGAG	126
VPA1348-R	GCTAAAAGCACCTGTTGGTAATA	
VP1549-F	CCAATCCAGCACAAGCCAT	98
VP1549-R	AGCAAAAACGCACGAAGC	
VP2925-F	GCAACACCAGCACCCATA	171
VP2925-R	CAAAAACGGCATCATCCAC	
VP2770-F	CATTTGGATGTTGTCGCCTG	198
VP2770-R	GCTAAGCCTGGTTCAATCACTC	
VP0819-F	TGTAATCGCCATTCGGTAG	182
VP0819-R	CCTTTTCAGTGGTTGGTTGTA	
VP0820-F	GAGATTCCGCTGGGTTTGTAA	103
VP0820-R	CCTGTGGCTTCTGCTGTGA	
VP2890-F	CGTCGCTCATCATCATTAGGTG	102
VP2890-R	CGAACAAAATCGTGGGCAT	
VPA1361-F	CTAACCACACAAGAAGCCAAC	130
VPA1361-R	GCTCGCAAGTGATGAGTAAT	
VPA1362-F	AGTTTTTGCCGCATCCAC	195
VPA1362-R	ATTATGAAATCAGCAGGGGT	
VP2516-F	TGTTGTCCGTCAGTTCTCG	201
VP2516-R	TGGTTAGTGCGGTTGGTAG	
VPA0606-F	CCATTGCGATGTGGCTCTG	122
VPA0606-R	ACACCGACGCTTCTACCCTT	
VP0301-F	CTGCTTTGGTTTATTTCTGG	193
VP0301-R	TAGGGCATCCTGCGTTAGT	
VP1995-F	ATCCGAAATCACCTTACCAT	115
VP1995-R	GTTCAATCAACTTCACGCTG	
VPA0660-F	CCTAACAGAGCGAGACGG	154
VPA0660-R	GCGACCAAAAGAGACCAGT	
VPA1436-F	CGCTACGCAACCCACATA	174
VPA1436-R	ACATACCACCGCCACCTTCT	
VP2467-F	ATACGGTGTTGGTTTCTGGGA	88
VP2467-R	TAGGTTGCTGCTGTCTTTATTTAC	
VPA0527-F	CATTTGCGAACGACTTTATC	144
VPA0527-R	CGTCTTTAGAACCTTGCCA	
RT-*tdh2*-F	CAACTTTTAATACCAATGCAC	129
RT-*tdh2*-R	GCCATTTAGTACCTGACG	

**Table 4:** Expression levels of *tdh2* and colonies of *V. parahaemolyticus* under stress.

Culture Condition	*tdh2* Expression Fold	Colonies (CFU/mL)
Control	1 ^a^	(900 ± 10) × 10^5 a^
8 h + 0.9% bile for 4 h	(1.32 ± 0.2) × 10^4 b^	(1 ± 0.5) × 10^5 b^
8 h + 4 °C for 4 h	(3.65 ± 0.4) × 10^2 c^	(170 ± 6) × 10^5 c^

The control was *V. parahaemolyticus,* which was cultivated in LBNB at 37 °C with shaking at 225 rpm/min for 12 h without cold and bile stress. The superscript letters (a, b, and c) represent significant differences (*p* < 0.05). The data were obtained in triplicate.

**Table 5:** The identified differential metabolites of WT and VP: △*tdh2* in LBNB at 37 °C for 12 h and their related pathways.

No.	Mz	Retention Time (min)	Identification	Pathway	Trend (VP: △*tdh2*/WT)
1	506.2478	8.35	PS (17:2 (9Z,12Z) /0:0)	Glycerophospholipid metabolism	↑
2	586.2769	10.14	PS (20:1 (11Z) /0:0)		↑
3	497.2874	11.75	PG (17:0/0:0)		↑
4	298.1201	1.03	2-Methylguanosine	Purine metabolism	↓
5	284.0987	1.34	Guanosine		↓
6	266.0863	1.43	Deoxyguanosine		↓
7	137.0466	0.92	Hypoxanthine		↓
8	282.1237	1.5	2′-O-Methyladenosine	-	↓
9	136.0617	0.84	Adenine	Purine metabolism	↓
10	243.0616	1.02	Uridine	Uridine monophosphate biosynthesis	↓
11	127.0518	1.77	Thymine	Pyrimidine metabolism	↓
12	455.0968	2.69	FMN	Riboflavin metabolism	↓

## Data Availability

The original contributions presented in this study are included in the article/Supplementary Material. Further inquiries can be directed to the corresponding authors.

## Supplementary Materials

The following supporting information can be downloaded at: https://www.mdpi.com/article/10.3390/microorganisms13081788/s1, Figure S1: RNA electrophoresis on 1% TAE agarose gel; Table S1: Total RNA concentration and purity.
