# Supplementary material for: The Toxin Gene tdh2 Protects Vibrio parahaemolyticus from Gastrointestinal Stress
Source: Microorganisms. 2025 Jul 31;13(8):1788. doi: 10.3390/microorganisms13081788 (PMC12388145; doi:10.3390/microorganisms13081788)
Supplement: Supplementary file 1 [file microorganisms-13-01788-s001.zip › microorganisms-3732457-supplementary.pdf]

Supplementary data

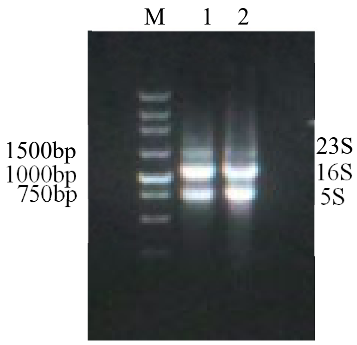

Figure S1 RNA electrophoresis on 1% TAE agarose gel; M.250 bp DNA makers; 1: WT; 2: VP*:* △*tdh2*.

Table S1 Total RNA concentration and purity

Strain	Concentration（μg/mL）	A_{260}/A_{280}	A_{260}/A_{230}
VP: △*tdh2*	625.62	2.30	1.98
WT	791.59	2.33	1.94

Note: That $1.9 < A_{260}/A_{230} < 2.1$ and $2.0 < A_{260}/A_{280} < 2\ 4$ indicates that RNA samples contain fewer impurities such as phenols, polysaccharides, and proteins.
